# Correction: Attrition in serum anti-DENV antibodies correlates with high anti-SARS-CoV-2 IgG levels and low DENV positivity in mosquito vectors—Findings from a state-wide cluster-randomized community-based study in Tamil Nadu, India

**DOI:** 10.1371/journal.pgph.0004157

**Published:** 2024-12-31

**Authors:** Sivaprakasam T. Selvavinayagam, Sathish Sankar, Yean K. Yong, Abdul R. Anshad, Samudi Chandramathi, Anavarathan Somasundaram, Sampath Palani, Parthipan Kumarasamy, Roshini Azhaguvel, Ajith B. Kumar, Sudharshini Subramaniam, Manickam Malathi, Venkatachalam Vijayalakshmi, Manivannan Rajeshkumar, Anandhazhvar Kumaresan, Ramendra P. Pandey, Nagarajan Muruganandam, Natarajan Gopalan, Meganathan Kannan, Amudhan Murugesan, Pachamuthu Balakrishnan, Siddappa N. Byrareddy, Aditya P. Dash, Vijayakumar Velu, Marie Larsson, Esaki M. Shankar, Sivadoss Raju

There is an error in affiliation 13 for author Pachamuthu Balakrishnan. The correct affiliation 13 is: Department of Research, Meenakshi Academy of Higher Education and Research (MAHER), Chennai 600078, India
