# Supplementary material for: Attrition in serum anti-DENV antibodies correlates with high anti-SARS-CoV-2 IgG levels and low DENV positivity in mosquito vectors—Findings from a state-wide cluster-randomized community-based study in Tamil Nadu, India
Source: PLOS Glob Public Health. 2024 Nov 21;4(11):e0003608. doi: 10.1371/journal.pgph.0003608 (PMC11581277; doi:10.1371/journal.pgph.0003608)
Supplement: S1 Table — (PDF) [file pgph.0003608.s002.pdf]

Supplemental Table 1: District-wise DENV positivity in mosquito pools in 2023

S.No	District	No. of mosquito pools		
		Tested	DENV positivity, n (%)	
1	Ariyalur	128	4	3·13
2	Chengalpattu	353	15	4·25
3	Chennai	302	22	7·28
4	Coimbatore	297	21	7·07
5	Cuddalore	460	12	2·61
6	Dharmapuri	217	11	5·07
7	Dindigul	425	9	2·12
8	Erode	427	26	6·09
9	Kallakkurichi	272	4	1·47
10	Kancheepuram	248	10	4·03
11	Kanniyakumari	284	13	4·58
12	Karur	267	3	1·12
13	Krishnagiri	626	17	2·72
14	Madurai	137	2	1·46
15	Mayiladuthurai	170	6	3·53
16	Nagapattinam	257	10	3·89
17	Namakkal	399	18	4·51
18	Perambalur	183	3	1·64
19	Pudukkottai	344	19	5·52
20	Ramanathapuram	448	24	5·36
21	Ranipet	246	11	4·47
22	Salem	834	43	5·16
23	Sivaganga	301	12	3·99
24	Tenkasi	434	10	2·30
25	Thanjavur	254	8	3·15
26	The Nilgiris	282	12	4·26
27	Theni	479	29	6·05
28	Thiruvallur	280	14	5·00
29	Thiruvarur	543	23	4·24
30	Thoothukkudi	582	41	7·04
31	Tiruchirappalli	338	12	3·55
32	Tirunelveli	329	9	2·74
33	Tirupathur	529	9	1·70
34	Tiruppur	673	14	2·08
35	Tiruvannamalai	31	1	3·23
36	Vellore	303	3	0·99
37	Villupuram	313	6	1·92
38	Virudhunagar	469	18	3·84
**Total**		**13464**	**524**	**3·76**
