# Supplementary material for: Attrition in serum anti-DENV antibodies correlates with high anti-SARS-CoV-2 IgG levels and low DENV positivity in mosquito vectors—Findings from a state-wide cluster-randomized community-based study in Tamil Nadu, India
Source: PLOS Glob Public Health. 2024 Nov 21;4(11):e0003608. doi: 10.1371/journal.pgph.0003608 (PMC11581277; doi:10.1371/journal.pgph.0003608)
Supplement: S2 Table — (PDF) [file pgph.0003608.s003.pdf]

Supplemental Table 2: District-wise distribution of DENV seropositivity

Sl. No.	District	Anti-DENV					Corrected			Force of infection (FOI)
		IgM +ve		IgG +ve		Total Sero +ve	IgM +ve %	IgG +ve %	Total sero +ve %	
		No.	%	No.	%					
1	Ariyalur	1	1·11	3	3·33	4	0·216	2·33	3·60	0·000957
2	Chengalpattu	8	4·44	40	22·22	38	3·598	23·94	22·67	0·0051
3	Chennai	24	13·33	43	23·89	63	13·768	25·85	38·56	0·08689
4	Coimbatore	14	5·19	22	8·15	32	4·445	7·84	12·07	0·002601
5	Cuddalore	7	3·89	8	4·44	14	2·962	3·60	7·41	0·001528
6	Dharmapuri	7	3·89	15	8·33	21	2·962	8·05	11·86	0·002363
7	Dindigul	2	1·11	6	3·33	21	0·216	2·33	11·86	0·002919
8	Erode	9	5·00	12	6·67	20	4·233	6·14	11·23	0·002617
9	Kallakkurichi	3	3·33	3	3·33	6	2·326	2·33	6·14	0·000779
10	Kancheepuram	1	1·11	2	2·22	3	0·216	1·06	2·33	0·000678
11	Kanniyakumari	3	1·67	7	3·89	10	0·420	2·96	4·87	0·001058
12	Karur	4	4·44	5	5·56	7	3·598	4·87	7·41	0·00176
13	Krishnagiri	13	7·22	5	2·78	17	6·776	1·69	9·32	0·002205
14	Madurai	36	13·38	9	3·35	45	13·825	2·34	17·65	0·00352
15	Mayiladuthurai	1	1·11	0	0·00	1	0·216	1·49	0·22	0·000238
16	Nagapattinam	4	4·44	3	3·33	7	3·598	2·33	7·41	0·001723
17	Namakkal	8	4·44	12	6·67	18	3·598	6·14	9·95	0·001988
18	Perambalur	2	2·22	4	4·44	6	1·055	3·60	6·14	0·001423
19	Pudukkottai	6	3·33	2	1·11	8	2·326	0·22	3·60	0·001033
20	Ramanathapuram	9	10·00	10	11·11	14	9·954	11·23	16·31	0·003676
21	Ranipet	5	5·56	4	4·44	8	4·869	3·60	8·68	0·001981
22	Salem	5	1·85	17	6·30	21	0·631	5·72	7·41	0·001542
23	Sivaganga	4	4·44	10	11·11	13	3·598	11·23	15·04	0·003319
24	Tenkasi	1	1·11	5	5·56	6	0·216	4·87	6·14	0·001604
25	Thanjavur	9	5·00	12	6·67	17	4·233	6·14	9·32	0·002045
26	The Nilgiris	2	2·22	6	6·67	8	1·055	6·14	8·68	0·001981
27	Theni	3	3·33	13	14·44	16	2·326	15·04	18·85	0·004255
28	Thiruvallur	3	1·67	12	6·67	14	0·420	6·14	7·41	0·001603
29	Thiruvarur	2	2·22	1	1·11	3	1·055	0·22	2·33	0·000634
30	Thoothukkudi	8	4·49	5	2·81	13	3·655	1·73	6·87	0·001597
31	Tiruchirappalli	6	3·33	6	3·33	10	2·326	2·33	4·87	0·001203
32	Tirunelveli	2	2·22	1	1·11	3	1·055	0·22	2·33	0·000699
33	Tirupathur	2	3·33	7	11·67	9	2·326	11·86	15·68	0·001687
34	Tiruppur	1	0·56	13	7·22	14	0·852	6·78	7·41	0·002000
35	Tiruvannamalai	1	0·56	21	11·67	22	0·852	11·86	12·50	0·002865
36	Vellore	5	4·17	4	3·33	9	3·280	2·33	7·09	0·001591
37	Villupuram	2	1·11	5	2·78	7	0·216	1·69	2·96	0·000872
38	Virudhunagar	6	3·33	3	1·67	7	2·326	0·42	2·96	0·000826
**Average**							**3·04**	**5·75**	**9·40**	**0·004141**

Supplemental Table 2 (continued)

Sl. No.	District	Anti-DENV titer		Force of infection		DENV cases
		IgM	IgG	FOI	no./1000 people/year	
1	Ariyalur	2·27	2·4	0·000957	0·96	81
2	Chengalpattu	2·74	9·42	0·0051	5·10	550
3	Chennai	4·80	10·48	0·08689	86·89	608
4	Coimbatore	4·52	11·31	0·002601	2·60	488
5	Cuddalore	3·42	4·78	0·001528	1·53	226
6	Dharmapuri	3·83	9·62	0·002363	2·36	128
7	Dindigul	4·24	8·57	0·002919	2·92	92
8	Erode	5·38	10·56	0·002617	2·62	70
9	Kallakkurichi	4·67	3·25	0·000779	0·78	144
10	Kancheepuram	2·13	5·3	0·000678	0·68	320
11	Kanniyakumari	3·16	9·03	0·001058	1·06	247
12	Karur	4·09	7·05	0·00176	1·76	19
13	Krishnagiri	5·35	8·39	0·002205	2·20	287
14	Madurai	5·19	7·31	0·00352	3·52	940
15	Mayiladuthurai	3·23	2·87	0·000238	0·24	83
16	Nagapattinam	3·27	5·13	0·001723	1·72	63
17	Namakkal	3·46	7·59	0·001988	1·99	209
18	Perambalur	2·27	6·28	0·001423	1·42	59
19	Pudukkottai	3·71	2·66	0·001033	1·03	134
20	Ramanathapuram	5·32	9·32	0·003676	3·68	68
21	Ranipet	3·57	5·4	0·001981	1·98	97
22	Salem	3·35	9·23	0·001542	1·54	139
23	Sivaganga	3·64	8·97	0·003319	3·32	175
24	Tenkasi	2·22	8·85	0·001604	1·60	266
25	Thanjavur	5·61	8·01	0·002045	2·05	22
26	The Nilgiris	2·82	5·44	0·001981	1·98	271
27	Theni	4·05	12·12	0·004255	4·26	340
28	Thiruvallur	3·13	2·45	0·001603	1·20	111
29	Thiruvarur	2·45	4·04	0·000634	1·60	712
30	Thoothukkudi	4·4	6·87	0·001597	0·63	601
31	Tiruchirappalli	2·85	6·6	0·001203	1·60	157
32	Tirunelveli	2·92	6·47	0·000699	0·70	266
33	Tirupathur	4·52	12·64	0·001687	3·46	140
34	Tiruppur	2·18	11·16	0·002000	1·69	139
35	Tiruvannamalai	2·76	10·11	0·002865	2·87	313
36	Vellore	3·12	5·67	0·001591	1·59	202
37	Villupuram	1·27	4·68	0·000872	0·87	207
38	Virudhunagar	3·92	7·44	0·000826	0·83	60
	**Average**	**2·94**	**8·1**	**0·004141**	**4·18**	
**Total**						**9034**
