# Supplementary material for: Attrition in serum anti-DENV antibodies correlates with high anti-SARS-CoV-2 IgG levels and low DENV positivity in mosquito vectors—Findings from a state-wide cluster-randomized community-based study in Tamil Nadu, India
Source: PLOS Glob Public Health. 2024 Nov 21;4(11):e0003608. doi: 10.1371/journal.pgph.0003608 (PMC11581277; doi:10.1371/journal.pgph.0003608)
Supplement: S3 Table — (PDF) [file pgph.0003608.s004.pdf]

Supplemental Table 3: District-wise distribution of SARS-CoV-2 seropositivity

Sl. No.	District	Samples (n)	Anti-SARS-CoV-2 IgG			
			No. IgG +ve	% IgG +ve	Mean	95% CI
1	Ariyalur	90	87	96·67	149·03	(132·87 - 165·19)
2	Chengalpattu	180	166	92·22	162·13	(151·87 - 172·39)
3	Chennai	180	154	85·56	151·57	(140·53 - 162·61)
4	Coimbatore	270	236	87·41	166·7	(158·94 - 174·46)
5	Cuddalore	180	165	91·67	176·79	(168·90 - 184·86)
6	Dharmapuri	180	153	85·00	170·46	(161·34 - 179·58)
7	Dindigul	180	172	95·56	160·17	(149·89 - 170·45)
8	Erode	180	165	91·67	157·57	(146·86 - 168·28)
9	Kallakuruchi	90	84	93·33	140·72	(140·91 - 174·23)
10	Kancheepuram	90	86	95·56	159·6	(143·76 - 175·44)
11	Kanyakumari	180	172	95·56	168·62	(159·26 - 177·98)
12	Karur	90	84	93·33	141·56	(125·26 - 157·86)
13	Krishnagiri	180	164	91·11	164·5	(155·22 - 173·78)
14	Madurai	269	247	91·82	182·24	(176·61 - 187·87)
15	Mayiladudhurai	90	77	85·56	155·31	(139·86 - 170·76)
16	Nagapattinam	90	86	95·56	176·57	(165·83 - 187·31)
17	Namakkal	180	158	87·78	166·18	(156·98 - 175·38)
18	Perambalur	90	84	93·33	168·86	(156·31 - 181·41)
19	Pudukottai	180	152	84·44	163·18	(153·34 - 173·03)
20	Ramanathapuram	90	79	87·78	178·11	(166·73 - 189·49)
21	Ranipet	90	80	88·89	171·78	(159·69 - 183·87)
22	Salem	270	228	84·44	169·58	(162·09 - 177·07)
23	Sivaganga	90	88	97·78	182·77	(173·08 - 192·46)
24	Tenkasi	90	84	93·33	166·17	(153·28 - 179·06)
25	Thanjavur	180	158	87·78	163·67	(153·56 - 173·78)
26	The Nilgiris	90	78	86·67	168·31	(155·37 - 181·26)
27	Theni	90	77	85·56	179·8	(168·43 - 191·17)
28	Thiruchirapalli	180	145	80·56	180·64	(162·81 - 180·31)
29	Thiruvallur	180	160	88·89	168·2	(158·86 - 177·54)
30	Thiruvarur	90	80	88·89	180·64	(169·47 - 191·81)
31	Thoothukudi	178	149	83·71	170·26	(161·05 - 179·47)
32	Tirunelveli	90	83	92·22	180·04	(169·24 - 190·84)
33	Tirupathur	60	47	78·33	172·38	(157·09 - 187·67)
34	Tiruppur	180	155	86·11	172·68	(164·01 - 181·35)
35	Tiruvannamalai	180	167	92·78	172·68	(164 - 181·35)
36	Vellore	120	97	80·83	174·64	(166·53 - 182·75)
37	Villupuram	180	155	86·11	160·57	(147·75 - 173·39)
38	Virudhunagar	180	160	88·89	165·07	(155·26 - 174·88)
**Total**			**4962**		**167·36**	
