# Supplementary material for: Attrition in serum anti-DENV antibodies correlates with high anti-SARS-CoV-2 IgG levels and low DENV positivity in mosquito vectors—Findings from a state-wide cluster-randomized community-based study in Tamil Nadu, India
Source: PLOS Glob Public Health. 2024 Nov 21;4(11):e0003608. doi: 10.1371/journal.pgph.0003608 (PMC11581277; doi:10.1371/journal.pgph.0003608)
Supplement: S4 Table — a. Mosquito pools positive for DENV and DENV seropositivity in the community. b. Mosquito positivity for DENV, and DENV positive cases. (PDF) [file pgph.0003608.s005.pdf]

Supplemental Table 4a: Mosquito pools positive for DENV and DENV seropositivity

Linear Regression				
**Outcome**	**Total DENV seropositivity**			
**Variable**	**Media**	**95% CI**		**P value**
Total population	43·13	31·11	55·16	<0·001***
Rural	28·96	13·28	44·64	<0·001***
Urban	18·34	11·55	25·13	<0·001***
Population density (10 units)	0·11	0·07	0·16	<0·001***
No. of mosquito clusters positive for DENV	2·7	-1·09	6·5	0·158
% of mosquito clusters positive for DENV	2·53	0·47	4·59	0·018*

Supplemental Table 4b: Mosquito positive for DENV and DENV positive case

Linear Regression				
**Outcome**	**Total DENV positive cases**			
**Variable**	**Media**	**95% CI**		**P value**
Total population	334·2	51·66	616·74	0·022*
Rural	50·04	-321·1	421·21	0·787
Urban	210·07	77·54	342·6	0·003
Population density (10 units)	0·02	0·02	0·02	<0·01**
No. of mosquito clusters positive for DENV	3·8	-2·55	10·14	0·234
% of mosquito clusters positive for DENV	23·34	12·6	59·28	0·02*
