# Supplementary material for: Attrition in serum anti-DENV antibodies correlates with high anti-SARS-CoV-2 IgG levels and low DENV positivity in mosquito vectors—Findings from a state-wide cluster-randomized community-based study in Tamil Nadu, India
Source: PLOS Glob Public Health. 2024 Nov 21;4(11):e0003608. doi: 10.1371/journal.pgph.0003608 (PMC11581277; doi:10.1371/journal.pgph.0003608)
Supplement: S5 Table — a. Factors associated with DENV seropositivity. b. Factors associated with DENV IgM and IgG levels. (PDF) [file pgph.0003608.s006.pdf]

Supplemental Table 5a: Factors associated with DENV seropositivity

Binary Regression								
**Outcome**	**Anti-DENV IgM positivity**				**Anti-DENV IgG positivity**			
**Variable**	**Media**	**95% CI**		**P value**	**Media**	**95% CI**		**P value**
Age	1	0·993	1·008	0·963	1	0·994	1·006	0·983
Gender	0·832	0·653	1·06	0·137	1·187	0·979	1·44	0·081
Vaccination status	1·291	0·899	1·854	0·167	1·185	0·903	1·556	0·221
AZD1222	0·996	0·629	1·578	0·987	0·814	0·581	1·141	0·233
BBV152	0·979	0·612	1·567	0·931	1·232	0·877	1·732	0·23
Anti-SARS-CoV-2 IgG (100 units)	0·811	0·634	0·936	0·044*	0·884	0·734	1·064	0·192

Supplemental Table 5b: Factors associated with DENV IgM and IgG levels

Linear Regression								
**Outcome**	**Anti-DENV IgM positivity**				**Anti-DENV IgG positivity**			
**Variable**	**Media**	**95% CI**		**P value**	**Media**	**95% CI**		**P value**
Age	-0·005	-0·024	0·014	0·599	-0·004	-0·019	0·012	0·651
Gender	-0·545	-1·163	0·072	0·084	-0·054	-0·553	0·445	0·832
Vaccination status	-1·227	-2·156	-0·298	0·01**	0·443	-0·307	1·194	0·247
AZD1222	0·108	-0·386	0·603	0·667	-0·226	-1·21	0·757	0·652
BBV152	-0·19	-0·691	0·311	0·458	0·194	-0·803	1·191	0·703
Anti-SARS-CoV-2 IgG (100 units)	-0·501	-1·03	-0·028	0·037*	0·398	-0·029	0·825	0·068
