# Supplementary material for: Attrition in serum anti-DENV antibodies correlates with high anti-SARS-CoV-2 IgG levels and low DENV positivity in mosquito vectors—Findings from a state-wide cluster-randomized community-based study in Tamil Nadu, India
Source: PLOS Glob Public Health. 2024 Nov 21;4(11):e0003608. doi: 10.1371/journal.pgph.0003608 (PMC11581277; doi:10.1371/journal.pgph.0003608)
Supplement: Attachment — Submitted filename: Response to Reviewers.docx [file pgph.0003608.s007.docx]

**Reviewer #1**

**Major comments**

**Comment 1:** Based on the review of the manuscript, it is evident that further information regarding the pre-analytical phase and sample preservation is essential to assess the study's internal validity accurately. The lack of details on these crucial aspects raises concerns about potential biases that could impact the study's outcomes. The title is misleading, and the authors did not provide any evidence that match the study title.

**Reply 1:** Details regarding the pre-analytical phase and sample preservation to assess the study's internal validity have now been revisited in the following responses, for clarity. The title has been modified as suggested, in line with the major conclusions arrived from the investigation.

**Comment 2:** Please clarify when the samples were collected and when they were processed to understand the preservation period. Furthermore, if possible, describe whether did you store controls to assess the samples preservation and the results of these assessments. If not, reflect this as a limitation in the discussion section.

**Reply 2:** Patient recruitment and sample collection was carried out during the period of Dec 2021 to Mar 2023. The blood samples were collected in serum separator tubes, packed and transported to the state public health laboratory in cold chain within 2 to 6 hours. The received samples were verified for integrity and stored at 2-8°C. The assays for DENV IgM and IgG were carried out within 4 hours of receipt. Quality control checks for the sample storage facility were assessed as part of the routine laboratory practices and the tests were found satisfactory.

**Comment 3:** Please provide further information on the pre-analytical phase, including sample collection procedures and handling to prevent biases. This additional information is crucial for assessing the reliability and accuracy of the study results.

**Reply 3:** The human blood samples were collected in serum separator tubes and transported to the laboratory within 6 hours. The sample integrity including triple layer package, cold chain, leakages, request forms and sender details are verified. The samples were stored at 2-8°C until testing. The assays for DENV IgM and IgG were carried out within 4 hours of receipt. The mosquito (adult and larva) samples collected from different localities were collected by the field workers and brought to the regional entomology laboratory for segregation of female Aedes mosquitoes. Vector discrimination was carried out at the regional entomology laboratory, as per the standard protocol. The selected mosquitoes are received by the state public health laboratory in cold chain within 18 hours of mosquito collection. The received mosquitoes were homogenized to collect the supernatant (described in the methods section). The samples were stored at -80°C until testing and were processed for RNA extraction and DENV PCR assay in batches (weekly).

**Comment 4:** Please explain in detail the measures taken to preserve serum samples and the duration between sample collection and processing.

**Reply 4:** Explained in the previous comment. All measures to preserve serum samples were taken including maintenance of cold chain, quality control of the storage facility and inclusion of test controls.

**Comment 5:** Please describe the steps taken to avoid antibody degradation prior to testing to ensure the accuracy of seroprevalence estimates.

**Reply 5:** The samples were transported to the laboratory within 6 hours of collection and hence exclusive test for antibody integrity was not required. There were minimal or no freeze-thaws before testing and proper storage and transport conditions were checked before accepting the sample for testing.

**Comment 6:** A word of caution about correlation is not causation unless you perform further analysis, so I will strongly recommend just limit your study to describe the trends and assessed using mathematical models.

**Reply 6:** Yes. As rightly mentioned by the reviewer, correlation does not necessarily imply causation. Hence we restricted with the term “associated/association” instead of “caused by” in the manuscript.

**Comment 7:** Please describe the statistical methods you used to test your study hypothesis about the “correlation” between the dengue seroprevalence and the aedine mosquito pools for dengue virus positivity during the COVID-19 pandemic.

**Reply 7:** Logistic regression analysis was carried out to identify factors associated with dengue seroprevalence. Correlation of the rate of DENV-positive mosquito pools with the rate of IgM, IgG and Total DENV seropositivity was carried out. Odds ratio was calculated for the associated factors with DENV seropositivity and DENV-positive human cases such as urban and rural population, population density and DENV-positive mosquito pools. Binary regression analysis was carried out to assess the factors associated with the anti-DENV IgM and IgG seropositivity including age, gender, SARS-CoV-2 vaccination, and type of vaccine.

**Comment 8:** Please review your method for correcting your prevalence estimates and explain why you are adding the “apparent prevalence” to the “specificity”.

**Reply 8:** The prevalence derived from apparent prevalence is considered to provide a more unbiased estimation (true positive and false positive rates) or true prevalence when added to the specificity (Habibzadeh et al. 2022).

**Comment 9:** Please double check your study period. You present different period of interest across the results that do not match the study methods.

**Reply 9:** The period of study was from Dec 2021 to Mar 2023. However, data from our on-going surveillance studies from 2016 to 2024 on DENV testing in mosquitoes were described in the manuscript for the comparison and trend analysis.

**Comment 10:** Please consider including a trend analysis

**Reply 10:** With respect, trend analysis is performed when data is collected longitudinal manner. In the present study, since the data is collected at a single time point, we use regression analysis to study the trend of outcome variable (viz. DENV seropositivity) control for various factors that affect the outcome variable.

**Comment 11:** Please be use a more conservative and objective approach to present your study results and conclusions and use the first paragraph of your discussion to present the main study results.

**Reply:** The first paragraph of the discussion is now presented with important findings of the study.

**Comment:** Please discuss your results instead about discussing unrelated issues.

**Reply 11:** The discussion has now been revisited adequately.

**Comment 12:** Please avoid misleading discussions about co-infection of DENV and SARS-CoV-2 because your study does not present any evidence about or against this issue.

**Reply 12:** The co-infection dynamics of DENV and SARS-CoV-2 are shown as a result of challenges such as inadequate surveillance and overlapping clinical presentation. The co-infection of the two viruses is discussed to emphasize the need for studying the seroprevalence of DENV along with the SARS-CoV-2.

**MINOR COMMENTS**

**Comment 13:** Please include the confidence interval of your estimates in your results and graphics.

**Reply 13:** The 95% CI (where appropriate) has been added to the results.

**Comment 14:** Seek a secondary review from a statistician with experience in seroprevalence studies to review your data analysis.

**Reply 14:** The data analysis was reviewed extensively by a statistician as suggested.

**Reviewer #2:**

**General:**

**Comment 1:** manuscript not line-numbered so that it is difficult to refer when reviewing.

**Reply 1:** The line numbers have been included in the revised manuscript

**Comment 2:** Authors should clearly refer to Figures in detail, including mentioning the alphabet on the Figure in the text (e.g. Fig 1A, Fig 1B, etc).

**Reply 2:** The Figure numbers are included now in the revised manuscript as per the reviewer’s suggestions

**Abstract:**

**Comment 3:**  please state clearly the aim of the study - what is the purpose of mentioning the binary and linear regression method in the Abstract? - is there conclusion in the Abstract?

**Reply 3:** The abstract has been modified as per the reviewer’s suggestions. The conclusion has now been included.

**Comment 4:** Please consider to use structured abstract

**Reply 4:** A structured abstract was not provided as per the journal’s submission guidelines.

**Method:**

**Comment 5:**  what type of Panbio IgM/IgG ELISA? Capture ELISA? Capture ELISA tends to use in acute infection/patients. The use of this ELISA in healthy subjects may underestimate the IgG seropositivity results. Author should use Indirect ELISA.

**Reply 5:** IgG Capture ELISA is considered to be more sensitive compared to indirect IgG ELISA. The cutoff value for the detection of IgG is set lower for indirect ELISA compared to capture ELISA. Indirect ELISA is used in the dengue seroprevalence cohort (Lopez et al. 2021) as a measure of past infection. However, use of capture ELISA has been reported to be valuable for seroprevalence in general healthy populations and for the differentiation of primary and secondary dengue (Velandia-Romero et al. 2020). In India, where Dengue is endemic, identification of high antibody titres is warranted (Kumar et al. 2021). Hence, we preferred to use capture ELISA which could help us identify past infection as well as secondary infections with high sensitivity and specificity. Studies on serosurveillance of dengue have been reported using capture ELISA (Tripathi et al. 2023). This has been included in the discussion section.

**Comment 6:** RT-PCR detection of DENV in mosquito is very tricky due to the presence of PCR inhibitors in the mosquito lysates. Does the method has been validated in mosquito samples? How to ensure that the RT-PCR does not falsely detect the Insect-Specific Flaviviruses that are very commonly presence in mosquito samples?

**Reply 6:** The RT-PCR based DENV RNA detection was carried out using commercial RT-PCR kit. Studies on detection of DENV RNA from the mosquito pools using the above kit was reported earlier (Kothandan et al. 2017). The assay kit is reported to be highly specific for DENV.

**Results:**
**Comment 7:**  what is the correlation of "the concurrent seroprevalence of dengue and SARS-CoV-2 in 2021" with mosquito samples assay? (it is difficult to refer the sentence when the manuscript is not line numbered!!!)

**Reply 7:** The seroprevalence of dengue and SARS-CoV-2 were concurrently studied along with mosquito pool testing for DENV. This is to test our hypothesis that the DENV circulation is hindered only at the community level in the tested population and DENV circulation in mosquitoes is not affected.

**Comment:** why NS1 is not used? Why only IgG/IgM>

**Reply:** NS1 will be detectable only within the first 5 days from the day of onset of illness (CDC guidelines). Furthermore, NS1 was not used because the study participants are not symptomatic. The study objective was to carry out seroprevalence of DENV and SARS-CoV-2 and correlate with DENV positive-aedine mosquito pools. NS1 is recommended only for diagnostic purposes and detection of acute infection, which was not in the scope of our study.

**Comment:**  the very high positivity rates of DENV in mosquito pools is somewhat doubtful. Typically, very low positivity rates is commonly observed when detecting DENV in mosquito samples. Authors must provide strong evidence that the high positivity rates is not caused by false-positive detection of Insect-specific Flaviviruses. It is strongly suggested that the authors isolate the virus or sequence the genome of the virus to proof that the detected DENV are truly from DENV.

**Reply:** In our study about 3.9% of samples were positive for DENV, which corroborated with previous studies in India that ranged from 1% – 5% DENV infection rate in Aedes mosquitoes (Sarma et al. 2023). Isolation of viruses was not attempted. However, representative DENV positive samples (from each administrative districts) are tested for DENV serotyping and sequencing of CprM region for confirmation and sequence analysis. The data are not shown as it was not part of the scope of this study. This was included in the discussion section.

**Comment:**  what are the serotype of the DENV detected in mosquitoes? - were positive controls (mosquito positive for DENV) used in the experiments?

**Reply:** Serotyping are performed for representative samples using seroptype-specific real time RT-PCR for epidemiological and public health data reporting purposes. The data is not shown here as it was not part of the scope of this study.

Discussion:
**Comment:**  COVID-19 pandemic has passed and many publication on dengue and COVID-19 are available. What is the novelty of this study? - Discussion is a bit more general and not specifically discuss the data. Authors should put more attention on the findings, its novelty, and the benefit of the finding.

**Reply:** The discussion section has been revised now.

**Conclusion:**
**Comment:**  it's not summarizing the finding but instead only stating about the importance of dengue and COVID-19.

**Reply:** Necessary changes in the manuscript have been carried out.

**Comment:**  so what is the main finding in brief?

**Reply:** The district-wise distribution of DENV-positive aedine mosquitoes and seroprevalence of DENV and SARS-CoV-2 was studied during the period of December 2021 to March 2023. The IgG, IgM seroprevalence and DENV-positivity in mosquito pools was low (6.45%, 3.9% and 3.96%, respectively) during the study period. The DENV-positive mosquito pool correlated with DENV-IgM and total seropositivity, but not with IgG. Factors including the total population, DENV-infested mosquito clusters and domiciliary status of participants (rural/urban) showed a significant correlation with DENV seroprevalence. Factors such as age, sex, vaccination status, type of vaccine administered and anti-SARS-CoV-2 antibodies were strongly associated with dengue seropositivity. The anti-SARS-CoV-2 IgG levels correlated significantly with dengue seropositivity, vaccination status correlated similarly with anti-DENV IgM. This indicates the possible implications of SARS-CoV-2 infection and subsequent vaccination on reduced DENV positivity in mosquitoes and seroprevalence in humans.

**Comment:**  what is the importance of this study?

**Reply:** The study shows the possible implications of SARS-CoV-2 infection and subsequent vaccination on reduced DENV positivity in mosquitoes and seroprevalence in humans.

**Reviewer #3**

**Comment** 1: The reasons attributed may not be conclusive. Another drawback of the study is that they did not cross check their findings using a random Dengue PRNT. The present reviewer could suspect less sensitivity and specificity of the Pan bio-ELISA IgM and IgG kit used for detecting the DENV seroprevalence, owing to the interference of SARS COV-2 virus infection (?) would have been a crucial factor in this drastic reduction of seropositivity on detecting Dengue virus antigen.

**Reply:** We humbly align with the reviewer’s view that the serological findings were not cross verified with Dengue PRNT. This was now included in the revised manuscript as a limitation. This would have been the most specific serological test for differentiating from other flavivirus infections. The authors however believe that the interference of SARS-CoV-2 is unlikely as the kit was validated for cross-reactivity with other flaviviruses and SARS-CoV-2 (Versiani et al. 2023).

**Comment** 2: However, Authors try to postulate many aspects and attributes to this drastic reduction in DENV seroprevalence to many factors, the major one being SARS COV-2 virus infection, without proof for any proposed concepts.

**Reply:** A historically low prevalence of dengue was reported in different countries during the outbreak of COVID-19. Of several factors analyzed, seroprevalence of SARS-CoV-2 also showed a significant correlation with dengue seropositivity indicating a possible impact of SARS-CoV-2 on dengue prevalence. The study lays serological evidence with a robust statistical analysis to demonstrate the possible correlation between the two viruses. As stated in the limitations, further experiments could prove the contribution of SARS-CoV-2 in the DENV prevalence.

**Comment 3:** Authors do not provide any references to their methodologies used, in the study.

**Reply:** References for methodologies were added appropriately in the revised manuscript.

**Comment 4:** Towards sampling of the population, authors calculate the sample size using a dengue seroprevalence rate to be 76.9%. The outcome results on the seroprevalence are 4.12% for IgM and 6.4% for IgG. An expert statistician has to verify, whether this sampling procedure had been correct, in the study!

**Reply:** A seroprevalence of 76.9% was considered based on the published literature (a population based serosurvey) in India. The study showed a high prevalence in all parts of India (Murhekar et al. 2019).

**Comment 5:** Some minor grammatical errors are there in the write up. May be corrected.

**Reply:** The manuscript has been thoroughly checked for language errors.

**Comment 6:** Introduction line 2. Aedes aegypti and Aedes albopictus should be written fully, instead of abbreviating those as Ae. aegypti and Ae. albopictus, when using species names for the first time in a write-up.

**Reply:** Necessary changes were carried out as suggested by the reviewer.
