# Supplementary material for: Attrition in serum anti-DENV antibodies correlates with high anti-SARS-CoV-2 IgG levels and low DENV positivity in mosquito vectors—Findings from a state-wide cluster-randomized community-based study in Tamil Nadu, India
Source: PLOS Glob Public Health. 2024 Nov 21;4(11):e0003608. doi: 10.1371/journal.pgph.0003608 (PMC11581277; doi:10.1371/journal.pgph.0003608)
Supplement: Attachment — Submitted filename: Reviewers comments and responses.docx [file pgph.0003608.s008.docx]

**Reviewers' Comments and Responses**

**Reviewer #1:** Thank you for responding to the review appropriately.

**Response#1:** Thank you for the recommendation.

----------------------------------------------

**Reviewer #2:** While authors have answered some of the concerns from reviewers, some important comments are not satisfactorily answered. The following points are very important for the data integrity and without further action from authors, the manuscript is not appropriate for publication:

**Query 1.** Authors do not agree to use indirect IgG ELISA which is more appropriate for sero-surveillance study.

**Response 1.** Thank you. Indeed, we do agree with the reviewer on the use of indirect IgG ELISA for sero-surveillance study. However, we conducted the sero-surveillance under the advice of public health experts who advocated the use of capture IgG ELISA citing that this has been employed previously in several dengue sero-surveillance studies. However, we concur with the reviewer that future sero-surveillance investigations shall be carried out with indirect IgG ELISA. We appreciate this observation and have mentioned this as one of the limitations of the study (line 426).

**Query 2.** Authors fail to prove that the very high positivity rates of DENV in mosquitoes is real and not because of non-specific amplification.

**Response 2.** The DENV real-time PCR was carried out using a validated commercial real-time PCR kit. Appropriate controls were used in the assay according to the manufacturer’s instructions and were found satisfactory. This has been mentioned clearly in the methods section of the manuscript.

**Query 3.** No serotype data is shown, as well as no genome sequence data to prove that the amplified DENV from mosquitoes are indeed from DENV, not from non-specific amplification/artefacts.

**Response 3.** Genome sequencing and serotyping was performed only for internal quality checks and were not shown in the manuscript. This has been described in the revised manuscript. Nonetheless, lack of serotype data has been listed as one of the limitations of the study.

--------------------------------

**Reviewer #3:** Authors have not addressed the comments carefully in the revised manuscript.
Still, I fear the conclusions arrived at IN THE MANUSCRIPT may put forth the misleading concept, that during covid pandemic Dengue incidence reduced drastically, without carrying out or citing any valid proof of concept scientifically.

**Query 1:** The drastic reduction of dengue incidence recorded may be due to the interference of SARS COV-2 infection preventing immunological detection of Dengue positives, as I have commented earlier.

As rightly mentioned by the reviewer, the reduction of dengue incidence could be due to the SARS-CoV-2 interference. The present study as stated earlier emphasizes serological correlation between the two viruses. However, the viral interference at the cellular or molecular level has not been performed. This is already been indicated as one of the limitations of the study. Kindly refer the publication by Cheng et al., 2022, "Antibodies against the SARS-CoV-2 S1-RBD cross-react with dengue virus and hinder dengue pathogenesis", Frontiers in Immunology, DOI: 10.3389/fimmu.2022.941923, which states that there is a clear interference of SARS COV-2 virus in the detection of Dengue cases by immunologically, as well as in the pathogenesis. I think this reported complex interactions, would have been the major attribute for the reduced incidence of Dengue detected using ELISA kits during the pandemic. Hence the conclusions could have been in these lines, possibly after carrying out a proof of concept study.

**Response 1.** As pointed out by the reviewer, we strongly agree with the reviewer. Hence, this further warrants an exclusive study on viral and immunological interference in disease pathogenesis.

**Query 2:** In case of selection of sample size also, there exist a clear error in the study. In the reply to comments you state that the sampling size was selected based on Murhekar et al, 2019, who comes out with an incidence of 76.9% for Dengue. But in your study which reports the incidence of Dengue to be about 6.4%. Thus the sample size you selected is an underrepresentation. You could have carried out a pre-test to estimate the sample size of the study.

**Response 2.** The study reported by Murhekar et al. 2019 on the burden of dengue indicate 76.9% among southern regions of India. The study carried out in 2017 included 5 states and three union territories. The study sampled four districts from each state – two each from rural and urban clusters respectively. In contrast, the present study sampled in 2021 was done using a randomized cluster sampling across all 38 districts of Tamil Nadu state, the sampling method has better representativeness compared to Murhekar et al. 2019. Therefore, it is not surprising that we observe the difference in the disease incidence. Disease prevalence could vary every year, but unbiased sampling method will ensure the true reflection of the parental population (Sauro et al. 2016; Representativeness and Randomness in Quantifying the User Experience, Second Edition, 2016). As suggested by the reviewer, the study could become a model on the dengue incidence and seroprevalence, which could be a valuable pre-test information for a large-scale study.

**Query 3:** Regarding xenomonitoring also, you have screened the samples by RT-PCR. You do not provide data on samples processed from different districts for years 2016-2023. In supplementary data you include only a table (Table S1) on the spatial distribution of mosquitoes sampled during 2023. Have you compared data through different years? Was the methodology of detection followed from 2016-2023 same?

**Response 3:** We regret that the spatial distribution for the previous years were not analyzed as the current study was directed to highlight the immunological correlation during post COVID-19 pandemic.

**Query 4:** You should have processed random samples using conventional PCR detection of CprM of E genetic markers of DENV and should have sequenced those to confirm the specificity and sensitivity of your detections.

**Response 4:** Yes. We have processed random samples using real-time PCR but not with conventional PCR targeting the CprM region. We have sequenced representative samples to confirm their sensitivity and specificity. This has been mentioned in the original submission.

**Query 5:** Besides study have many limitations, a least a few could have been avoided to make study more scientific.

**Response 5:** We concur with the reviewer. We have endeavoured our best to rationalise the findings with valid available methods in this part of the developing world. We have attempted to address the gaps in knowledge and potential lacunae have been briefed as limitations. Thank you for the suggestions that we believe have advanced our knowledge and have improved the manuscript.
